# Modulation of cyclic nucleotide-mediated cellular signaling and gene expression using photoactivated adenylyl cyclase as an optogenetic tool

**DOI:** 10.1038/s41598-017-12162-4

**Published:** 2017-09-21

**Authors:** Meenakshi Tanwar, Lohit Khera, Nemneineng Haokip, Rajeev Kaul, Aruna Naorem, Suneel Kateriya

**Affiliations:** 1 Department of Biochemistry, University of Delhi South Campus, Benito Juarez Road, New Delhi, 110021 India; 2 Department of Microbiology, University of Delhi South Campus, Benito Juarez Road, New Delhi, 110021 India; 3 Department of Genetics, University of Delhi South Campus, Benito Juarez Road, New Delhi, 110021 India; 4 School of Biotechnology, Jawaharlal Nehru University, New Delhi, 110067 India

## Abstract

Cyclic nucleotide signaling pathway plays a significant role in various biological processes such as cell growth, transcription, inflammation, in microbial pathogenesis, etc. Modulation of cyclic nucleotide levels by optogenetic tools has overcome certain limitations of studying transduction cascade by pharmacological agents and has allowed several ways to modulate biological processes in a spatiotemporal manner. Here, we have shown the optogenetic modulation of the cyclooxygenase 2 (Cox-2) gene expression and their downstream effector molecule (PGE_2_) in HEK-293T cells and the development process of *Dictyostelium discoideum* via modulating the cyclic nucleotide (cAMP) signaling pathway utilizing photoactivated adenylyl cyclases (PACs) as an optogenetic tool. Light-induced activation of PACs in HEK-293T cells increases the cAMP level that leads to activation of cAMP response element-binding protein (CREB) transcription factor and further upregulates downstream Cox-2 gene expression and their downstream effector molecule prostaglandin E2. In *D. discoideum*, the light-regulated increase in cAMP level affects the starvation-induced developmental process. These PACs could modulate the cAMP levels in a light-dependent manner and have a potential to control gene expression and their downstream effector molecules with varying magnitude. It would enable one to utilize PAC as a tool to decipher cyclic nucleotide mediated signaling pathway regulations and their mechanism.

## Introduction

Cells respond to the external stimuli using complex molecular signaling cascades for transmitting information from the external environment into the cell. Most of the signaling pathways use secondary messengers to convey information. Adenosine 3′, 5′-cyclic AMP (cAMP), a universal secondary messenger, is employed by diverse organisms to control various biological processes. cAMP triggers a myriad of cellular reactions using a network of intracellular signaling pathways via phosphorylation of specific target proteins. cAMP-mediated signaling has been implicated in various physiological processes ranging from intermediary metabolism, gene expression, neuronal signaling, cellular proliferation and apoptosis, contractility and relaxation of heart^1–4^. The synthesis of cAMP from ATP is catalyzed by adenylate cyclase. Adenylate cyclases work as central relay stations which receive and amplify first signals such as hormones, alternations in ionic components, change in pH in the environment with the change in the amplitude of cAMP^5^. The regulatory effects of cAMP in eukaryotes are mediated via various downstream effector proteins such as Protein kinase A (PKA)^6^, Exchange Protein directly Activated by cAMP (EPAC)^7^, Cyclic Nucleotide-gated ion channels (CNG)^8^. These effector molecules sense the changes in intracellular cAMP levels and regulate numerous cellular responses^9–12^. Moreover, the amplitude and duration of cAMP levels in the cell is also governed by phosphodiesterase (PDE) enzymes which are responsible for the breakdown of cAMP^13^. The major outcome of cAMP signaling is mediated through protein kinase A (PKA). The specificity of PKA in mediating cAMP signaling is provided by A-kinase-anchoring proteins (AKAPs) by targeting the PKA to specific substrates, effectors and subcellular compartments^14^. It also provides the versatility by assembling with multiprotein signaling complexes (signal terminators and components of other signaling pathway) allowing termination of signal and cross talk between different signaling pathways^15^. In eukaryotes, cAMP molecules bind to the regulatory subunits of the PKA complex releasing the catalytically active kinase subunit, which further phosphorylates large number of cytosolic and nuclear proteins. PKA regulates various specific cellular biological functions such as gluconeogenesis, glycogen synthesis, glycolysis, lipogenesis, intestinal secretion, ion conductance, collecting duct functions, cell survival pathway etc. and intracellular signaling pathway (for example calcium signaling, mitogen-activated protein kinase signaling, Rho signaling, T cell receptor signaling) via phosphorylating the substrate (enzymes/protein) involved in the respective cellular pathway^16,17^. Regulation of gene expression by PKA is accomplished via activation of cAMP response element binding protein (CREB) transcription factors. The activation of CREB via phosphorylation will initiate the activation of transcription factors and thus expression of the specific downstream genes^9^. Another effector protein EPAC, also indirectly regulates the activation of transcription factor CREB via Ca2^+^/calmodulin kinase II (CaMKII) pathway. Varied cellular processes have been linked to CREB activity such as development of learning and memory^18,19^ metabolic regulation such as gluconeogenesis^20^, lipogenesis^21^, immune regulation^22^, and control of cell growth and survival^23,24^. The genome wide analysis confirms the expression of cAMP-dependent target genes in response to rise in the level of cAMP^25^. Expression of numerous genes involved in metabolism, cell cycle and secretory pathways are also regulated by various extracellular signals and elevation in the cAMP level via CREB transcription factors^26,27^. For instance, the CREB also governs the expression of Cox-2 gene, which is implicated in the survival of cells^26^ and in various pathophysiological processes such as fever, ischemia, cancer and Morbus Alzheimer^28^. Upregulation of the Cox-2 gene promotes prostaglandin E2 (PGE_2_) synthesis and suppresses apoptosis and finally leads to tumor progression. Conversely, Cox-2 inhibition promotes apoptosis and inhibits cell proliferation^29^. Besides these functions, cAMP acts as both extracellular and intracellular secondary messenger in *D. discoideum* and is thus a critical molecule in the development of social, haploid free-living amoebae that feed on soil bacteria. The starvation-induced developmental process in *D. discoideum* requires extensive cAMP signaling with the well-defined spatiotemporal patterning of factors, resulting in aggregation of unicellular amoebae into a fruiting body^30,31^. As an extracellular messenger, cAMP is required in nanomolar concentrations in a pulsatile manner for cell aggregation and also for repression of prestalk gene expression^32–35^. As an intracellular signal, cAMP controls prespore gene expression and germination of spores. The primary target of intracellular cAMP is protein kinase A, which is required for both prespore and prestalk differentiation^36–39^. Cyclic nucleotide signal transduction is precisely choreographed in a highly spatial and temporal manner to control gene activity and coordinate complex, intricate network in a cell.

To gain insights into signaling pathways under various physiological processes and unhealthy conditions, it is imperative to study its control on the signaling cascade and their downstream genes. Pharmacological agents like agonist activating adenylyl cyclases^40^ and PDE inhibitors^41^ have been used to examine signal transduction to decipher the decision-making process of the cells signaling. Due to lack of spatial and temporal control in these approaches, in decoding the dynamic information in intracellular signal transduction requires new tools. Optogenetics, a newly emerging field, which enables the control of signaling molecules or protein activity by simply illumination^42–46^. The photoreceptors (light-sensitive proteins) are used as a tool for optogenetic studies that respond to light and affect the cellular responses. Depending on the photoreceptors, optogenetic tools respond to different wavelength of light and pass signals to downstream effector molecules. Photoactivated adenylyl cyclases (PACs) are BLUF based photoreceptors coupled with adenylyl cyclases and are used to modulate cAMP levels in the cell in response to blue light. Several reports used PACs for modulating ion currents in oocytes of *Xenopus laevis* ^47^, altering cAMP levels for behavioral study in *Drosophila* ^47,48^ and finding the mechanism underlying cAMP-dependent axonal morphogenesis^49^.

In this report, we demonstrated the optogenetic control of cAMP-mediated CREB-dependent gene expression and its downstream effector molecules. Our approach harnesses the photoactivated adenylyl cyclases from amoeboflagellate protozoa, *Naegleria gruberi* (NgPACs) and bacteria *Beggiatoa* (bPAC). The *N. gruberi* genome has four PACs (NgPAC1, NgPAC2, NgPAC3, and NgPAC4) genes^50^. We employed the PACs to manipulate the cAMP-signaling pathway and analyzed the cAMP-mediated CREB-dependent gene expression in response to blue light. We showed that the different PACs exhibited light regulated adenylyl cyclase activity, which in turn could control the activation of the CREB transcription factor leading to the expression of the downstream gene (Cox-2) and their associated downstream effector molecule (PGE_2_) in HEK-293T cells. We have also employed PACs as an optogenetic tool in modulating cAMP level in *D. discoideum*. We observed that starvation-induced development was affected in cells expressing PACs, as a result of alteration in the cAMP level in response to light.

## Results

### Expression and light regulated cyclase activity of PACs in mammalian (HEK-293T) cells

**Figure 1:**
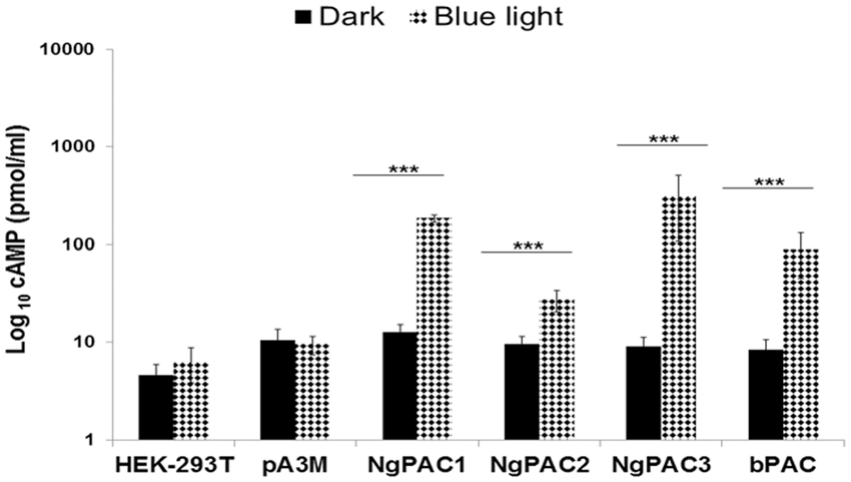
Light induced activation of the PACs in mammalian (HEK-293T) cells. The total cAMP level in the HEK-293T cell lysates expressing NgPAC1, NgPAC2, NgPAC3, bPAC and empty vector pA3M in the dark and after irradiation with blue light (460 nm). ***p < 0.0005 measured by student’s *t* test between dark and light conditions. Error bars represents the mean of S.E.M.

For examining the effect of light-induced adenylyl cyclase activity on cAMP-dependent gene expression and the downstream signaling pathway, transient heterologous expression of PACs (NgPACs from *N. gruberi* and bPAC from *Beggiatoa*) were performed in mammalian cells. For expression in human cell lines, codon optimized synthetic genes, NgPAC1, NgPAC2, NgPAC3, and bPAC, were cloned into the pA3M mammalian expression vector (using primers mention in Table S1) and expressed in HEK-293T cells as a C-terminally tagged myc tag fusion protein (PAC::myc). Immunoblotting with an anti-myc antibody showed that all PACs (NgPAC1, NgPA2, NgPAC3, and bPAC) displayed robust expression in HEK-293T cells (Fig. S1). For assessment of the photoactivation of PACs in the HEK-293T cells, a competitive immunoassay was executed to quantify the cAMP level in the lysate of cells expressing PACs kept in the dark as well as after blue light irradiations. No change in the cAMP level was observed in control HEK-293T and HEK-293T cells transfected with empty pA3M vector upon blue light illumination compared to dark (Fig. 1). However, HEK-293T cells expressing different PACs exhibited adenylyl cyclase activity with elevated levels of cAMP upon photoactivation of PACs with blue light, as compared to levels in the dark (Fig. 1). Strong light regulated elevation in the cAMP level was displayed by NgPAC1 (∼14 fold), NgPAC3 (∼34 fold) and bPAC (∼10 fold). In contrast, NgPAC2 exhibited a moderate increase in cAMP levels upon photoactivation indicating NgPAC2 has a weak photoactivated adenylyl cyclase activity in HEK-293T cells. Further, PACs showed moderate cyclase activity in the dark (basal cyclase activity). These results confirmed the expression of PACs and the light regulated adenylyl cyclase activity in mammalian (HEK-293T) cells.

### Optogenetic control of the phosphorylation of CREB transcription factor

**Figure 2:**
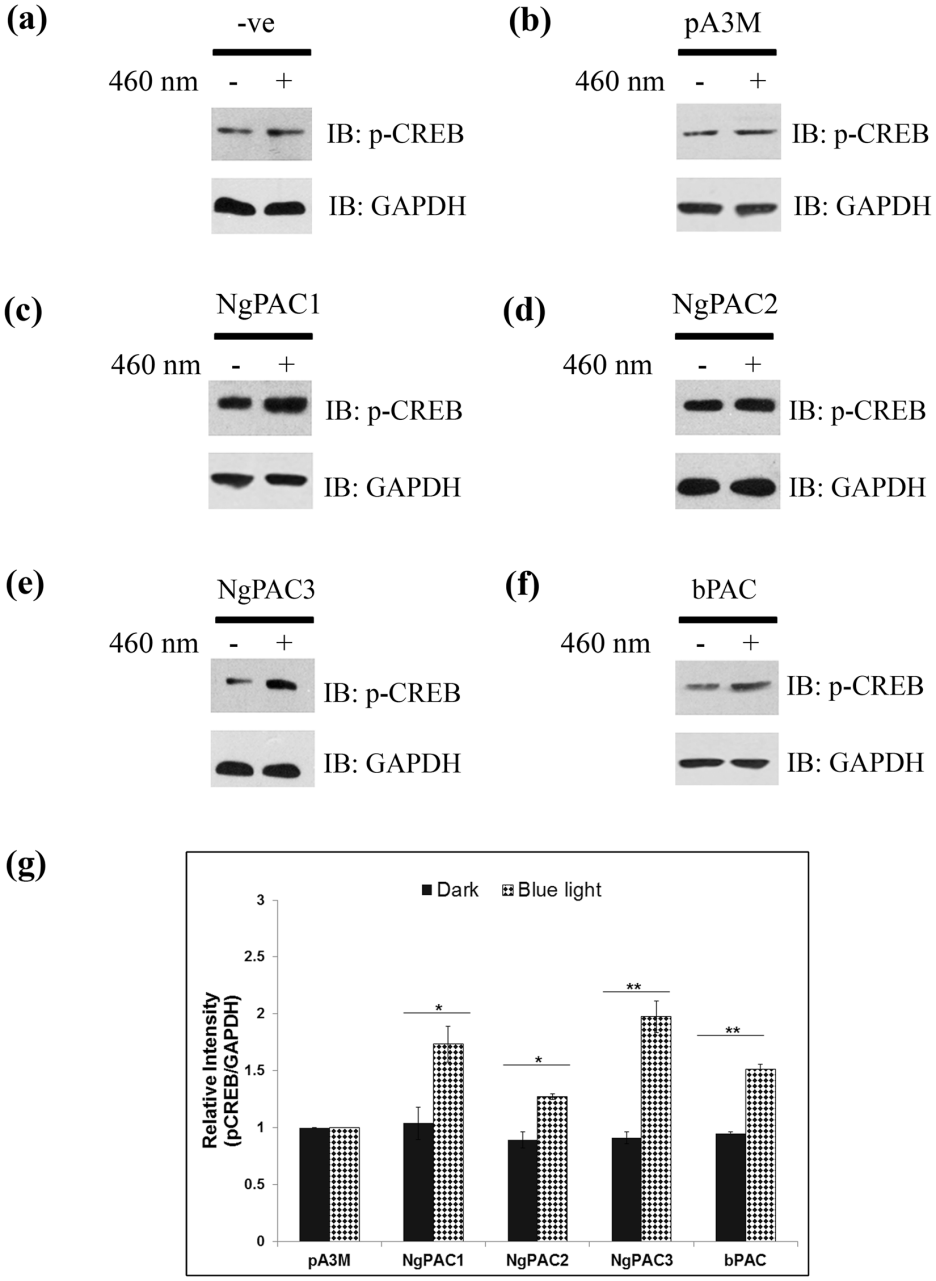
Optogeneticaly induced phosphorylation and activation of the CREB in PACs expressing mammalian (HEK-293T) cells. Western blot showing phosphorylated CREB level (upper panel) and GAPDH (lower panel) in the total cell lysate of (**a**) HEK‐293T cells, HEK-293T cells expressing (**b**) empty pA3M vector (**c**) NgPAC1, (**d**) NgPAC2, and (**e**) NgPAC3 (**f**) bPAC, in the dark (−) and after blue light illumination (+). pA3M expressing HEK‐293T cells was served as a negative control. (**g**) The relative intensity of phospho CREB:GAPDH in each sample was determined by densitometry. The relative intensity of phospho CREB:GAPDH in negative control cells was arbitrary set as one and the value achieved for PACs expressing cells were expressed as fold induction. The results are the average of the three independent experiments. The full length blots are presented in supplementary section (Fig. S8).

Alterations in cAMP levels in the cell are translated into various intracellular effects by cAMP-binding proteins. cAMP-dependent protein kinase A (PKA) and the directly activated exchange proteins (EPAC) are the two main effector proteins that mediate the changes in cAMP to downstream signaling cascades. PKA phosphorylates target proteins in the nucleus it activates the CREB transcription factor by phosphorylating serine 133 to induce expression of its downstream target genes^51^. EPAC also regulates the phosphorylation of CREB transcription factor (at Ser133) by activation of calmodulin kinase II (CaMKII)^52,53^ through mobilization of intracellular Ca^2+^ via a pathway that involves the phospholipase C (PLC) and Ca^2+^/calmodulin kinase II (CaMKII)^54^. In order to investigate light-induced activation of CREB in HEK-293T cells expressing different PACs (NgPAC1, NgPAC2, NgPAC3, and bPAC), immunoblotting experiments were executed using antibodies against phosphorylated CREB and GAPDH. A control experiment with only HEK-293T and HEK-293T cells transfected with empty vector (pA3M) showed no significant change in the phosphorylation level of CREB after blue light irradiation (Fig. 2a,b). Cells expressing different PACs showed an increase in the phosphorylation level of CREB when subjected to blue light in comparison to the cells incubated in the dark (Fig. 2c–f). Densitometric analysis of the relative phosphoserine CREB levels with the loading control GAPDH was performed and the results depicted in a bar chart showing the corresponding average of three independent experiments (Fig. 2g). Densitometric analysis revealed that phosphorylated CREB was elevated in the cells expressing NgPAC3, NgPAC1, and bPAC upon exposure to light. The increase in the level of phosphorylated CREB was only slightly higher in the cells expressing NgPAC2 after illumination. Immunoblot and densitometric analysis of total CREB level in the cells expressing PACs revealed no significant increase in the total CREB level with GAPDH upon illumination with blue light (Fig. S2). The photoactivation of PACs under blue light led to the increased level of phosphorylated or activated CREB in HEK-293T cells.

### Optogenetically regulated CREB alters CREB regulated Cox-2 gene expression

**Figure 3:**
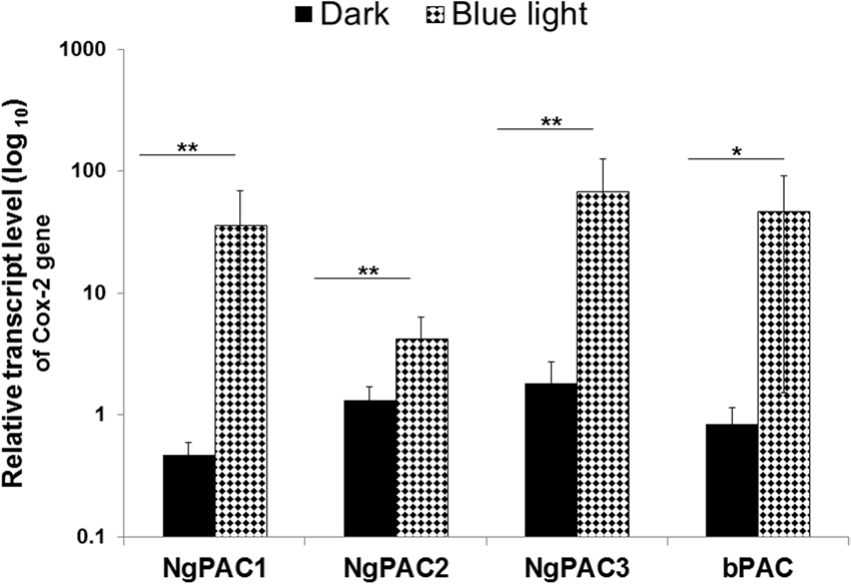
Light induced regulation of the Cox-2 gene expression via modulating cAMP signaling pathway in PACs expressing mammalian (HEK-293T) cells. Real‐time PCR assessment of Cox‐2 mRNA levels (ΔΔCt values) from HEK-293T expressing PACs (NgPAC1, NgPAC2, NgPAC3 and bPAC) cells kept in the dark (solid black bar) and exposed to blue light (460 nm) (filled bar). GAPDH mRNA level was used as an internal control. ***p < 0.0005, **p < 0.005, measured by student’s *t* test between dark and light conditions. Error bar represents the mean of S.E.M, n = 4 experiments in duplicate.

One way cAMP affects biological processes is by changing the expression of the downstream target genes. In such cases, CREB transcription factors act as a principal mediator for alteration in the expression of genes including Cox-2. Various factors like cytokines, mitogenic and stress inducing factors influence the expression of Cox-2 genes^55^. To assess whether optically, activated CREB could control the expression of the Cox-2 gene, its mRNA level was quantified by real-time PCR. The effect of blue light on Cox-2 mRNA levels in HEK-293T cells (untransfected) was assayed, which suggested no significant change in expression in the dark and after illumination treatment (Fig. S3). The mRNA level of Cox-2 in the cells expressing PACs were normalized with the mRNA level of GAPDH (calculate ΔCt value). Further, mRNA levels of Cox-2 in HEK-293T cells expressing PACs were normalized with the mRNA level of Cox-2 in cells transfected with empty pA3M vector (calculate ΔΔCt value). The relative Cox-2 mRNA level was found to be augmented upon illumination with blue light in the HEK-293T cells expressing PACs (Fig. 3). The highest Cox-2 expression upon photoactivation was observed in the cells expressing NgPAC3. Approximately same levels of Cox-2 gene expression were shown by NgPAC1 and bPAC upon photoactivation and was minimum in the cells expressing NgPAC2. However, NgPAC1 expressing cells showed greatest fold increase in Cox-2 gene expression, followed by bPAC, NgPAC3, and NgPAC2 expressing cells. The results showed that the optically activated CREB transcription factor could augment the expression of Cox-2 gene in HEK-293T cells (Fig. 3).

### Optogenetically regulated Cox-2 gene expression is reflected by downstream effector PGE2 levels

**Figure 4:**
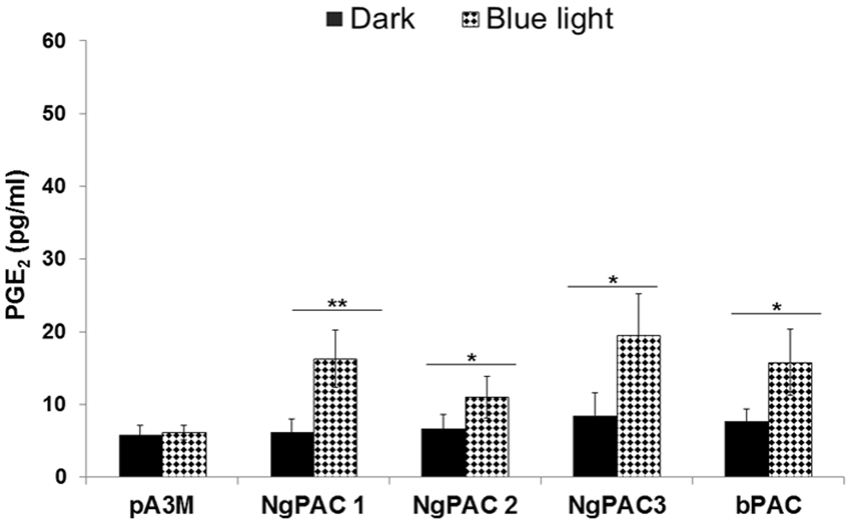
Optogeneticaly induced upregulation of the Cox-2 regulate the downstream PGE_2_ effector molecule in PACs expressing mammalian (HEK-293T) cells. The PGE_2_ concentration was determined in the supernatant of HEK-293T cells transfected with empty vector (pA3M) or PACs (NgPAC1, NgPAC2, NgPAC3 and bPAC) in dark and after treated with blue light (460 nm). Data from dark-adapted cells are displayed as solid bar; data from illuminated cells are shown as filled bar. ***p < 0.0005, **p < 0.005, measured by student’s *t* test between dark and light conditions. Error bars represents the mean of S.E.M, n = 4 experiments in duplicate.

Cox-2 controls production of PGE_2_ a metabolic product of arachidonic acid that is involved in various physiological functions such as regulation of immune response, gastrointestinal integrity, cell survival, as well as associated with pathological conditions like tumorigenesis, chronic inflammation, Alzheimer’s disease^56,57^. Different types of cancers including colon cancer, lung cancer, and gastric cancer have been reported to be associated with overexpression of Cox-2 and increased production of downstream effector molecule PGE_2_^58–61^. Optically activation of Cox-2 can lead to increased levels of the downstream effector PGE_2_ molecule, therefore the expression pattern of PGE_2_ was determined in dark and upon blue light illumination using immunoassay. For the control experiment, no increase in PGE_2_ amounts was observed in HEK-293T cells transfected with empty vector (Fig. 4). It was found that after illumination with blue light the level of PGE_2_ in the supernatant collected from cells expressing different PACs was increased, as compared to the cells kept in the dark (Fig. 4). The maximum level of PGE_2_ in magnitude upon photoactivation was found in NgPAC3. Approximately the same level of PGE_2_ was observed in cells expressing NgPAC1 and bPAC. While NgPAC2 expressing cells displayed lowest level of PGE_2_ after photoactivation. However, the maximum fold increase in PGE_2_ levels upon photoactivation was by cells expressing NgPAC1, followed by cells expressing NgPAC3, bPAC, and NgPAC2. These results suggested that the optically activated CREB transcription factor could regulate Cox-2 gene expression thereby leading to an increase production of PGE_2_ in HEK-293T cells.

### Light regulated cyclase activities of PACs in *D. discoideum* transformants

**Figure 5:**
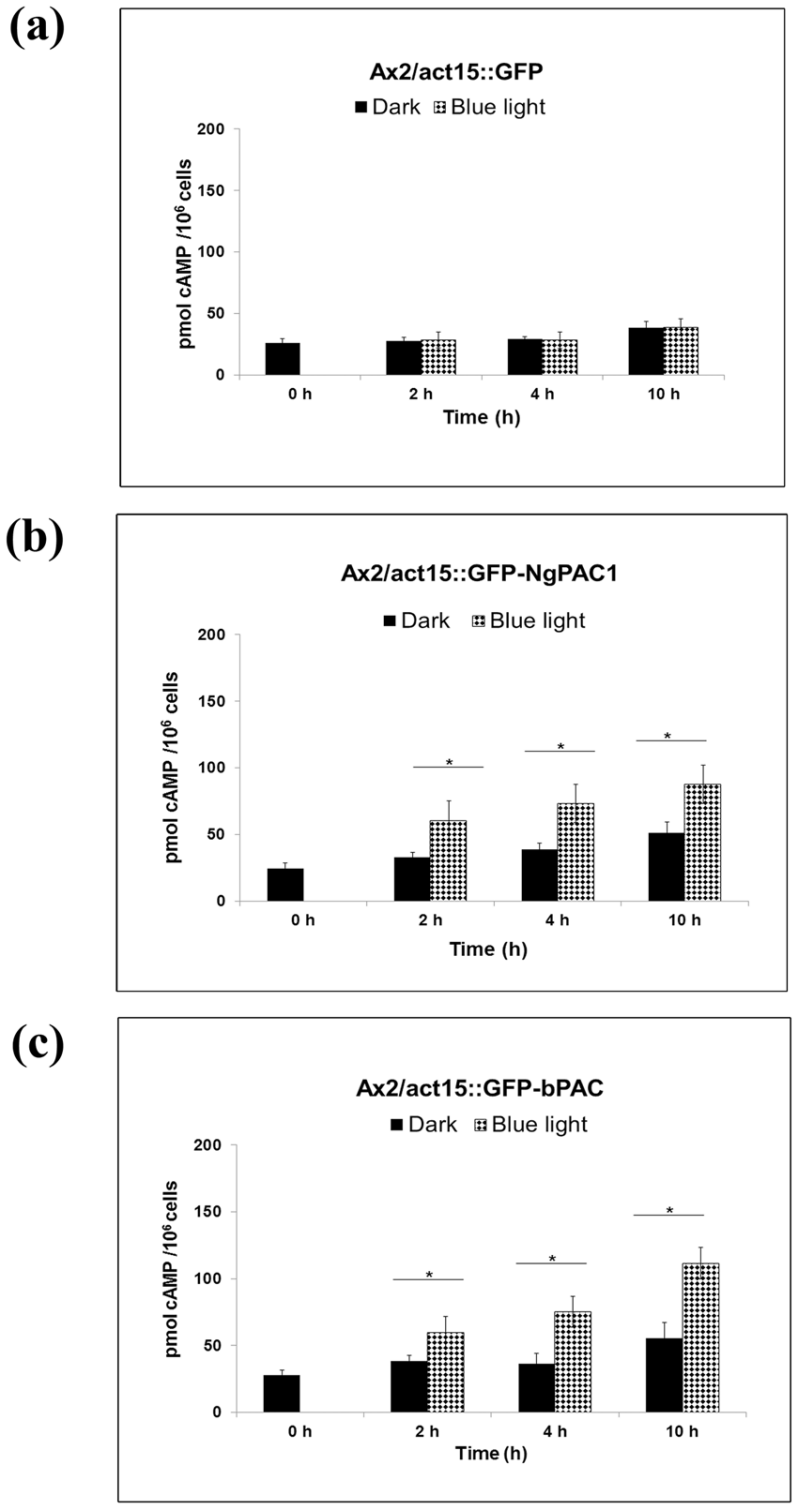
Light regulated adenylyl cyclase activity of PACs in vegetative *D. discoideum transformants*. The cAMP level in vegetatively grown cell lysates of (**a**) Ax2/act15: GFP (control) (**b**) Ax2/act15: GFP‐NgPAC1 and (**c**) Ax2/act15: GFP‐bPAC were analysed. Cells grown in the dark and in the presence of blue light for different time periods (0, 2, 4, 10 h) were harvested and lysed. The cAMP level was measured using ELISA in the lysates of each transformed cell lines and plotted against time. Light induced activation of PACs resulted in the elevation of cAMP level (p < 0.05) in Ax2/act15:: NgPAC1 and Ax2/act15:: bPAC cells compared to controls kept in the dark. Error bar represents the S.E.M.

cAMP act as both an extracellular and an intracellular messenger in controlling the differentiation and development of *D. discoideum* ^31^. NgPAC1, and bPAC genes were cloned in frame with green fluorescent protein (GFP) tag under constitutively active actin 15 promoter (act15:: GFP-PAC) in pTX-GFP vector. The PAC constructs were transfected into Ax2 cells, and the expression of each GFP, GFP-NgPAC1, and GFP-bPAC fusion proteins was confirmed by western blotting with mouse α-GFP antibodies in the respective transformants. As reported earlier^62^ a band of 40 kDa corresponding to GFP was detected only in the control sample, i.e., Ax2 cells transfected with vector (Ax2/act15:: GFP), but the corresponding band was absent in non-transfected Ax2 cells (Fig. S4). As expected, western blots confirmed the expression of different PACs as monoclonal α-GFP antibodies detected different GFP-PAC fusions in individual Ax2 transformants. Analysis using confocal microscopy also showed the expression of both the GFP-NgPAC1 and GFP-bPAC fusions in the respective Ax2/act15:: GFP-PAC transformants. No GFP fluorescence was observed in Ax2 cells (Fig. S5a). Confocal analysis showed strong fluorescence in Ax2/act15:: GFP cells confirming the cytoplasmic localization of GFP in the cell (Fig. S5b). Similar fluorescence was also observed in the cytoplasm of Ax2/act15:: GFP-NgPAC1 and Ax2/act15:: GFP-bPAC cells indicating the localization of GFP-NgPAC1 and GFP-bPAC fusion proteins in the cytoplasm of transfected cells (Fig. S5c,d). To determine the expression of NgPAC1 and bPAC in Ax2/act15:: GFP-NgPAC1 and Ax2/act15:: GFP-bPAC cells, we examine light regulated cyclase activity of NgPAC1 and bPAC in vegetatively growing cells. The cells were cultured in the dark, and in the presence of blue light for the different times and harvested at 0 h, 2 h, 4 h, and 10 h. The cAMP levels in the cell lysate were determined by immunoassay. No change in the level of cAMP was observed in Ax2/act15:: GFP cells in the presence of blue light (Fig. 5a). It was found that the cell lysates of Ax2/act15:: GFP-NgPAC1 and Ax2/act15:: GFP-bPAC displayed an increase in cAMP level upon photoactivation of PACs when grown in the presence of blue light (Fig. 5b,c), as compared to the lysate of these cells grown in the dark. However, the observed cAMP levels in vegetatively growing Ax2/act15:: GFP-PAC transformants was found to be low. It might be due to the activity of cAMP–phosphodiesterases in vegetative cells induced by increased level of cAMP^63^. These results suggested that the expression of PACs and their light regulated cyclase activity could control the cAMP level in *D. discoideum*.

### Activation of PACs in *D. discoideum* leads to alteration in developmental process

**Figure 6:**
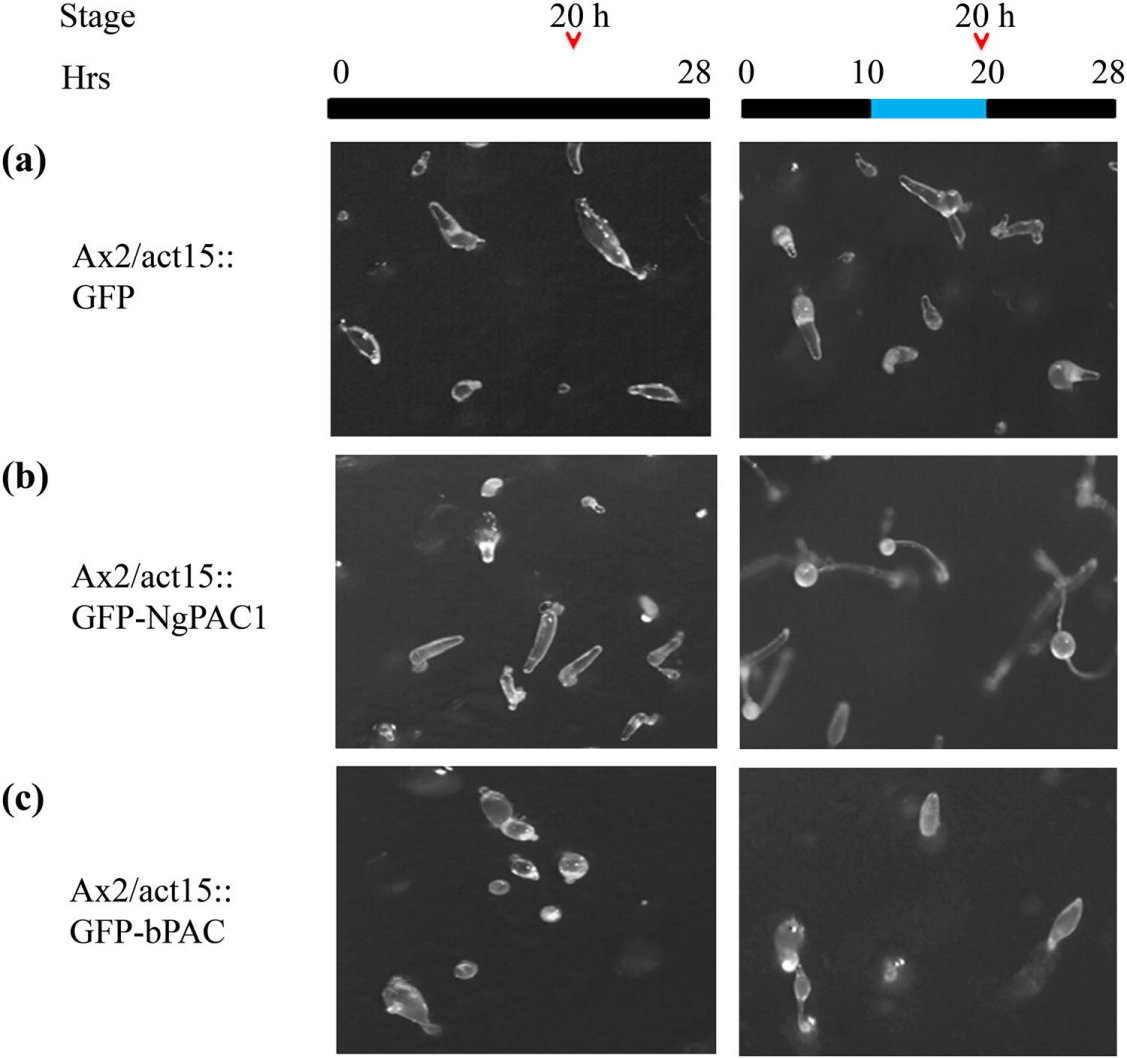
Effect of blue light induced activation of PACs on *D. discoideum* development at 20 h. Phenotype of (**a**) Ax2/act15:: GFP, (**b**) Ax2/act15:: GFP-NgPAC1, (**c**) Ax2/act15:: GFP-bPAC cells at 20 h of development stage, when developed on KK_2_ non-nutrient agarat a density of 1 × 10^6^ cells/cm^2^. The plates were kept in the dark for first 10 h (black bar) after which the plates were exposed to blue light for next 10 h (blue bar). Developmental stages at 20 h were scored and photographed at the time as indicated by open arrow head.

**Figure 7:**
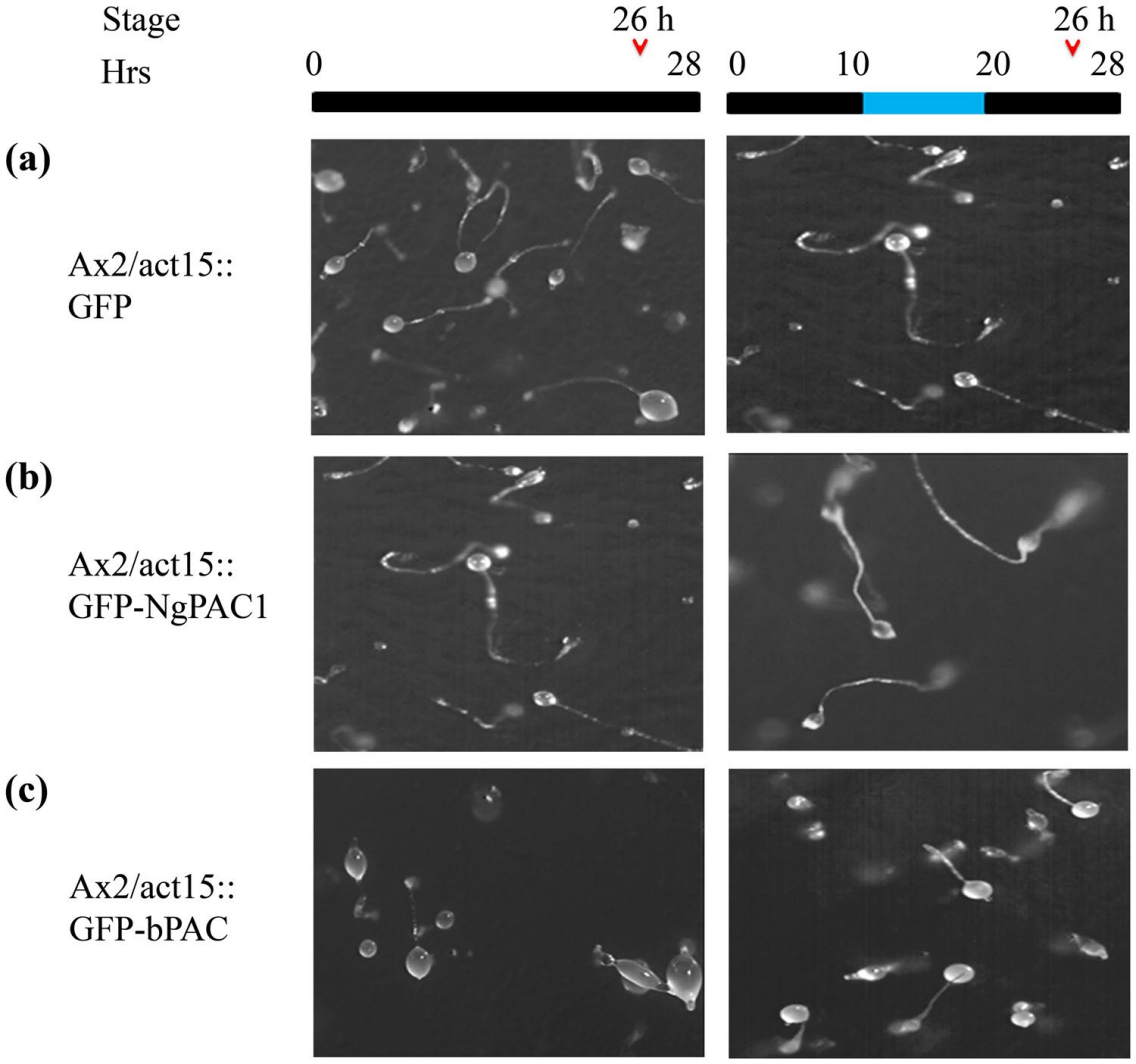
Effect of photoactivation of PACs on *D. discoideum* development at 26 h. Development of (**a**) Ax2/act15:: GFP, (**b**) Ax2/act15: GFP-NgPAC1, (**c**) Ax2/act15: GFP-bPAC at 26 h, when grown on non‐nutrient KK_2_ agar plates at a density of 1 × 10^6^ cells/cm^2^. For development, the cells on plate were incubated in dark for 10 h. For photoactivation of PAC, the cells were then irradiated with blue light for next 10 h and then incubate again in dark at 22 °C. The developmental stages were recorded and photographed as indicated by open arrow head at 26 h.

We next extended the optogenetic approach to assess modulation in the developmental behavior of *D. discoideum*. A developmental study of Ax2, Ax2/act15:: GFP, Ax2/act15:: GFP-NgPAC1 and Ax2/act15:: GFP-bPAC cells were performed on non-nutrient agar and incubated in the dark. Ax2/act15:: GFP cells showed normal developmental morphology similar to the wild-type Ax2 cells. Ax2/act15:: GFP cells formed aggregates by 10 h (Fig. S6a). The aggregates were followed by the formation of tipped mound and a migratory slug and culminants by 20 h (Fig. 6a). Development was completed by 24–26 h with the formation of fruiting bodies (Fig. 7a). For activation of PACs, the cells were incubated in the presence of blue light after 10 h of development. Similar growth profile were observed for Ax2 (Fig. S7a,b) and Ax2/act15:: GFP cells (Figs 6a and 7a) in dark and after illumination with blue light. Similarly, Ax2/act15:: GFP-NgPAC1 cells when developed in the dark formed aggregates by 10 h (Fig. S6b), followed by formation of tipped mound and slug at 20 h (Fig. 6b, Table. S3) and fruiting bodies formation by 26 h (Fig. 7b, Table. S3). Surprisingly, Ax2/act15:: GFP-NgPAC1 cells showed precocious development (more than 60% structures) when illuminated with blue light (Fig. 6b, Table. S3) by forming fruiting bodies at 20 h as compared to vector control which formed fruiting body at 26 h. Interestingly, as compared to Ax2/act15:: GFP cells, Ax2/act15:: GFP-bPAC cells exhibited delayed development in the dark forming aggregates at 10 h (Fig. S6c), tipped mound by 20 h (Fig. 6c, Table. S3) and by 26 h major fractions of multicellular structures are at culminants stage (Fig. 7c, Table S3). However, blue light illuminations of Ax2/act15:: GFP-bPAC cells for 10 h–20 h during development lead to normal development compared to the control cells (Ax2/act15:: GFP) by the formation of culminant structures by 20 h (Fig. 6c, Table. S3) and fruiting bodies (more than 60% structures) at 26 h (Fig. 7c, Table. S3). These results demonstrated that the optical control of cAMP levels might result in the alteration of cAMP-mediated signaling which could, in turn, affect the developmental process in *D. discoideum*.

## Discussion

In recent years, optogenetic studies have been reported using photoactivated adenylyl cyclase (PACs) as an optogenetic tool to perturb signaling pathways to get insights into the diverse roles of cAMP in various cellular processes. The first photoactivated adenylyl cyclase (PAC) reported was euPAC from *Euglena gracilis* and consisted of two BLUF followed by cyclase domain to mediate photo avoidance in the organism^64^. Subsequently, PACs described from *Beggiotoa* ^48^ and *N. gruberi* ^50,65,66^ are smaller than euPAC and composed of single BLUF and cyclase domains. PACs (NgPAC1, NgPAC2, NgPAC3, and bPAC) exhibit typical BLUF photochemistry with varying recovery kinetics from signaling state to dark state after photoactivation with blue light. It also showed *in vitro* light-induced cyclase activity with cyclase activities variable in the amplitude^48,50,65,66^. EuPAC and bPAC have been used for modulation of cAMP level in cells^47,48,67^. Due to requirement of different speeds of cAMP-based biochemical operations to execute different functions in different cells and tissues, there is a prerequisite to look for more tools with varying recovery kinetics and cyclase activities for modulation of cAMP cascades in a spatial manner in different cells and tissues.

Here, we have described the functional characterization of photoactivated adenylyl cyclases (PACs) from *N. gruberi* for able to control the cAMP-mediated gene expression in mammalian (HEK-293T) cells and the multicellular development of *D. discoideum* in a light-dependent manner. To assess diverse prospects of PACs in regulating cAMP signaling, PACs genes were successfully expressed in mammalian (HEK-293T) cells and *D. discoideum*. Photoactivation of PACs leads to an increase in the level of cAMP in both HEK-293T (Fig. 1) and *D. discoideum* (Fig. 5) cells, which results in modulation of cAMP-dependent gene expression in mammalian (HEK-293T) cells (Fig. 3) and in changes in development in *D. discoideum* (Figs 6, 7).

Expression of PACs in mammalian (HEK-293T) cells revealed that the cyclase activity is light-regulated upon photoexcitation. All NgPACs (NgPAC1, NgPAC2, and NgPAC3) and bPAC exhibited different light-regulated cyclase activities in the HEK-293T cells (Fig. 1). Induction of cAMP upon photoactivation of PACs with blue light led to the activation of CREB by phosphorylating serine 133 (Fig. 2). Activation of CREB after PAC photoactivation results in the upregulation of Cox-2 mRNA levels indicating the possible control of gene expression in a light-dependent manner (Fig. 3). NgPAC3 showed maximum induction of Cox-2 gene expression in terms of amplitude. Of all the PACs, NgPAC1 showed the highest fold increase in Cox-2 gene expression, followed by bPAC, NgPAC3, and NgPAC2. The pattern of upsurge in Cox-2 gene expression was different from the increase in the phosphorylation of CREB. This might be due to crosstalk between intracellular signaling pathways that are linked by cAMP^68^, or due to other signaling pathways that regulate the expression of the Cox-2 gene. It has been reported that mitogen-activated protein kinases (MAPKs) also regulate the Cox-2 mRNA levels both at transcriptional and post- transcriptional level^69^. The light-induced upregulation of Cox-2 expression also led to increased expression of its downstream effector PGE_2_ (Fig. 4). All PACs showed elevation in PGE_2_ upon photoactivation. Among all PACs, NgPAC3 showed maximum induction of PGE_2_ upon photoactivation. All PACs led to variable increases in PGE_2_ after blue light illumination (Fig. 4) that would modulate PGE_2_ levels at different amplitudes. Modulation of expression of Cox-2 and downstream PGE_2_ levels via a cAMP-mediated CREB driven transcriptional program by Nexrutine, a *Phellodendron amurense* bark extract and TGF-β2 has also been reported^70,71^. PACs could be a useful tool for optical regulation of Cox-2 in particular compartment via targeting with a specific signal sequence and in a temporal manner in order to study the effect of their upregulation on other molecules and gene expression and could open a way for identification of therapeutic targets for diseases where cAMP is involved.

The functional expression of PACs in *D. discoideum* vegetative cells demonstrated the light regulated adenylyl cyclase activities of the PACs (Fig. 5). Activation with blue light of PACs expressed by *D. discoideum* resulted in an altered developmental phenotype (Figs 6, 7). Surprisingly, the cells expressing PAC (bPAC) when kept in the dark displayed delayed development, as compared to control cells expressing vector only (Ax2/act15:: GFP cells). This delayed phenotype could be due to disruption in pulsatile signaling of cAMP required for cell aggregation^72,73^. Activation of PACs after 10 h of development in Ax2/act15:: GFP-NgPAC1 cells (Fig. 6b) displayed advanced development, as compared to the cells developed in the dark as well as the cells expressing vector only (Ax2/act15:: GFP) (Fig. 6a). Although bPAC expressing Ax2/act15:: GFP-bPAC cells (Figs 6c and 7c) showed delayed development in the dark as compared to control cells (Ax2/act15:: GFP) (Figs 6a and 7a), while the development was found to be similar to the Ax2/act15:: GFP cells in the presence of blue light (Figs 6c and 7c). Nonetheless, the developmental phenotype either advanced development in Ax2/act15:: GFP-NgPAC1 cells under blue light, or delayed developmental phenotype in Ax2/act15:: GFP-bPAC cells in the dark might be due to alterations in the intracellular level of cAMP due to activities of PACs. An earlier study has also reported that an increase in intracellular cAMP levels triggered the culmination process, which is actively controlled by the cAMP-dependent protein kinases^74^. Recently, a PAC composed of LOV and cyclase domains (known as mPAC) was shown to restore development of *D. discoideum* cells lacking an adenylate cyclase A *(aca* ^*−*^ *)* in a light-dependent manner^75^.

In the view of applications in research, recovery kinetics of photoreceptors is an important criterion. It represents the time required for a photoreceptor to come back from signaling state to ground state. Biophysical studies demonstrated the fast recovery kinetics of bPAC^48^ and NgPAC1^65^, while slower recovery kinetics of NgPAC2^66^ and NgPAC3^50^ have been reported. These PACs with varying recovery kinetics would enable researchers to manipulate the cAMP levels for brief or extended times. In addition to changing time, the different cyclase activity of PACs (bPAC, NgPAC1, NgPAC2, and NgPAC3) could modulate cAMP levels with varying amplitude in different cells and tissues that require different degrees of cAMP. The different cAMP fluxes lead to control gene expression and their downstream molecules at different level. It suggests that one PACs might not be suitable to study all cAMP-related pathways. The different levels of cAMP, in turn, would be able to modulate the various pathways that are controlled by the cAMP effector molecules (PKA, EPAC, CNG). PKA has higher sensitivity for cAMP than EPAC and can be activated by nanomolar cAMP^7^, while CNG and EPAC are maximally active at higher cAMP concentrations of 10 μM and 40 μM, respectively^76^. The diverse PAC might allow one to study in detail the pathways mediated by different cAMP effector molecules by modulating cAMP.

The presented results, in combination with earlier biophysical and biochemical data on NgPACs^50,65,66^ and bPAC^48^, uphold the promise of PAC’s optogenetic potential (PACs with varying recovery kinetics and cyclase activities) to differentially manipulate the cyclic nucleotide mediated signaling cascade and gene expression in the cells. Additionally, these various PACs could be used as an optogenetic tool corresponding to the requirement of the cAMP level for the signal transduction pathway in the cells. Molecular components of signaling cascade are excellent targets for developing the new therapeutic strategies. Use of PAC could be a promising tool for both basic and medical research in studying the cAMP signaling pathways and could provide a tool for investigating the targets for treatment of cancer, in respiratory disease, in various infections and pathophysiological processes where cAMP signaling pathway is involved^77–82^. The application of the PAC could also be extended to understand the complex networking of cAMP signaling pathways.

## Materials and Methods

### Cell culture and transfections of mammalian HEK-293T cells

Human embryonic kidney HEK-293T cells were grown in Dulbecco’s modified minimum Eagle’s medium (DMEM, PAN Biotech, Aiden bach, Germany) supplemented with 5% (v/v) heat-inactivated fetal bovine serum, 1% L-glutamine (PAN Biotech), 1% penicillin, streptomycin and amphotericin (PAN Biotech) at 37 °C with 5% CO_2_. For transient expression, HEK-293T cells were transfected in 100 mm dish with 10 μg plasmid DNA (pA3M, NgPAC1-pA3M, NgPAC2-pA3M, NgPAC3-pA3M and bPAC-pA3M) using polyethyleneimine (PEI), linear molecular weight 25,000 (Polyscience, Inc. Warrington, PA, USA). For transfection, DNA was added to the 150 mM NaCl followed by addition of PEI in the mixture. The mixture was incubated at room temperature for 15 min and then added to the HEK-293T cells. The media was changed after 3 h of transfection.

### Cell illumination for activation of PACs, lysis and immunoblotting of HEK-293T cells lysate

For light related experiments, all illumination was performed after 48 h of transfection using blue LED (460 nm, 25 µW/cm^2^) in a self-built lightbox. Cell lysis was carried out after illumination, by removing media and adding lysis buffer (50 mM Tris-Cl, 150 mM NaCl, 1% trigitol, pH-7.5) supplemented with protease inhibitors (pepstatin 1 μg/ml, leupeptin 1 μg/ml, aprotinin 1 μm/ml, PMSF 100 μM) (Sigma, Aldrich, St. Louis, Missouri, USA). After cell lysis, the lysate was centrifuged at 15,000 × g for 5 min at 4 °C. The protein samples were prepared by mixing the obtained supernatant of the samples with 2 × SDS Laemmli buffer. The equal amounts of protein were resolved on SDS-PAGE, transferred to nitrocellulose membrane and immunoblotted with anti-myc, anti-phosphorylated CREB (Cell Signaling Technology, Inc. Massachusetts, USA) antibody. The same membrane was stripped using stripping buffer (62.5 mM Tris-Cl pH-6.8, 2% sodium dodecyl sulphate and 100 mM β-mercaptoethanol) and again immunoblotted with GAPDH antibody (Cell Signaling Technology, Inc. Massachusetts, USA).

### *In vitro* cyclase activity of the PACs in PAC expressing HEK-293T cells

Immunological quantification of the cAMP level was performed to determine the light regulated adenylyl cyclase activity of PACs in PAC expressing HEK-293T cells. Light regulated adenylyl cyclase activity of PACs were measured in transiently PAC expressing HEK-293T cell lysate at room temperature in assay buffer (50 mM Tris-Cl pH-7.5, 1 mM MgCl_2_, 1 mM DTT) supplemented with 1 mM IBMX containing 60 μg of protein. The cyclase reaction was initiated by the addition of 100 μM ATP under two conditions: dark and illuminate with blue light (460 nm, 50 µW/cm^2^) using blue LED (Conrad, Luxeon lll Emitter LXHLPB09, Germany). The reaction was stopped after 15 min by addition of 50 μl of 0.1 M HCl. cAMP level in the above reactions was determined by a competitive immunoassay using an ELISA kit (Cayman, Michigan, USA), according to the instructions of the manufacturer.

### Relative quantification of the Cox-2 transcript by real-time PCR

For quantification of CREB regulated Cox-2 gene expression upon illumination with blue light, total RNA was extracted from HEK-293T cells transfected with plasmids (pA3M, NgPAC1-pA3M, NgPAC2-pA3M, NgPAC3-pA3M and bPAC-pA3M respectively) using PureLink RNA mini kit (Ambion, Life technologies, Foster City, CA, USA) as per the manufacturer’s instruction. RNA was reverse transcribed with random hexamers and SuperScript™ II Reverse Transcriptase (Invitrogen, Carlsbad, USA) according to the instructions laid by manufacturers. The resulting cDNA was amplified by Real-time PCR in duplicate with the use of a USB VeriQuestTM SYBR Green qPCR master mix (Affymetrix, Santa Clara, CA, USA) according to the manufacturer’s instructions, in a 7900HT Fast Real-Time PCR Detection System (Applied Biosystem, Foster City, CA, USA). The primers used for real-time PCR were Cox-2 and GAPDH (Table S2). The conditions employed for real-time PCR was: 2 min at 50 °C, 10 min at 95 °C, 40 cycles of denaturation at 95 °C for 15 s and annealing/extension at 60 °C for 1 min. The GAPDH housekeeping gene was used to normalize samples for DNA quality and quantity. Relative mRNA levels of Cox-2 gene were determined by ΔΔCt approach, as suggested by Applied Biosystems for the relative quantification of mRNA transcripts.

### *D. discoideum* cell culture and stable transformants expressing PACs

*D. discoideum* (Dd) Ax2 strain was used in all the experiments. *D. discoideum* cell culture, transformation and maintenance were done as described previously^83^. Cells were cultured axenically in standard HL5 medium on petridishes or alternatively in association with *Klebsiella. aerogenes* at 22 °C. For transformation of PAC genes in Ax2, approximately 5 × 10^6^ cells were harvested, washed and resuspended in electroporation buffer (E buffer: 50 mM sucrose, 10 mM Na_2_HPO_4_, 10 mM KH_2_PO_4_). The cells were electroporated in a 0.1 cm cuvette at 0.65 kV and 0.1 ms time constant using BIORAD MicroPluserTM (Bio-Rad Laboratories, Inc., Hercules, California, USA). Positive transformants were selected in HL5 medium supplemented with 10 μg/ml G418 (Geneticin; Biobasic). The stable transformants were propagated in the HL-5 medium containing 20 μg/ml G418.

### Fluorescence confocal microscopy imaging of *D. discoideum* transformants expressing PACs

Ax2 transformants expressing PAC (Ax2/act15:: GFP-NgPAC1, Ax2/act15:: GFP-bPAC), were screened for GFP expression by fluorescence confocal microscopy. Ax2 cells expressing GFP (Ax2/act15:: GFP) and different GFP-PAC fusion proteins (Ax2/act15:: GFP-NgPAC1, Ax2/act15:: GFP-bPAC) were harvested by centrifugation at 200 × g for 5 min. The cells were washed with KK_2_ buffer and stained with DAPI (0.2 μg/ml) for 5 min. After staining, the cells were briefly washed with KK_2_ buffer, resuspended in 1X PBS and mounted on glass slides. The cells were visualized for both DAPI and GFP fluorescence. The images were captured on Leica TCS SP5 confocal microscope (Leica, Germany) at central instrumentation facility (CIF), University of Delhi South Campus. The images were processed on Leica image processing software provided by the manufacturer.

### Preparation of *D. discoideum* total soluble protein fraction and immunoblotting

Cells from 20 ml culture of Ax2 transformants (Ax2/act15:: GFP, Ax2/act15:: GFP-NgPAC1, AX2/act15:: GFP-bPAC) were pelleted by centrifugation at 200 × g for 5 min. The cells were lysed in lysis buffer (50 mM Tris-Cl pH-7.5, 50 mM NaCl, 2 mM MgCl_2_, 0.5 mM EDTA, 0.05% Triton X-100, 200 mM PMSF) by incubation on ice for 1 h. The cell lysate was centrifuged at 12,000 × g for 15 min at 4 °C. The supernatant was collected and boiled for 5 min in SDS-PAGE sample laemmli buffer. The proteins were separated on SDS-PAGE (12%), transferred onto nitrocellulose membrane and probed with primary GFP antibody (Santa Cruz Biotechnology, Inc, Texas, USA) and secondary horseradish peroxidase (HRP) conjugated mouse secondary antibody (Sigma-Aldrich, St. Louis, Missouri, USA).

### Cellular cAMP level in vegetatively growing *D. discoideum* transformants

Vegetatively grown Ax2 cells were used for measuring the cellular cAMP level. Ax2 transformants cells were grown in flasks in the dark and in the presence of blue light. Cells (5 × 10^6^) were harvested at 0 h, 2 h, 4 h and after 10 h of growth by centrifugation at 200 × g for 5 min. The cells were washed with HL5 medium and lysed in lysis buffer and incubated on ice for 30 min. All cyclase reactions were terminated by adding 50 μl of 0.4 M EDTA, followed by boiling for 2 min. The boiled lysate was centrifuged and the supernatant was used for assaying the cAMP level using ELISA (Cayman, Michigan, USA), according to the manufacturer’s instruction.

### Multicellular development of *D. discoideum* transformants

Multicellular development of cells was induced by plating cells on non-nutrient agar i.e KK_2_ agar (1.5% agar in 4 mM K_2_HPO_4_, 16 mM KH_2_PO_4_ pH-6.5). Exponentially grown cells were harvested, washed twice with KK_2_ buffer and plated on KK_2_ agar plate at a density of 1 × 10^6^ cells/cm^2^. After proper drying, the cells were allowed to develop in the dark at 22 °C. Photographs were taken at the different time point under stereozoom microscope to score development stages. For illumination, experiments initially cells were developed in dark for 10 h, then the cells were illuminated with blue light (460 nm, 25 µW/cm^2^) using a blue LED (Conrad, Luxeon lll Emitter LXHL-PB09, Germany) for 10 h and further allowed to develop in dark at 22 °C.

### Data analysis and statistics

All data are reported as means ± SEM with significance denoted as *p < 0.05, **p < 0.01, ***p < 0.001. Statistical analyses (*t* test) were accomplished with Graphpad Prism 5.0 software.

## Electronic supplementary material

Supplementary information
