# Supplementary material for: Modulation of cyclic nucleotide-mediated cellular signaling and gene expression using photoactivated adenylyl cyclase as an optogenetic tool
Source: Sci Rep. 2017 Sep 21;7:12048. doi: 10.1038/s41598-017-12162-4 (PMC5608697; doi:10.1038/s41598-017-12162-4)
Supplement: Supplementary file 1 — Supplementary information [file 41598_2017_12162_MOESM1_ESM.doc]

**Modulation of cyclic nucleotide-mediated cellular signaling and gene expression using photoactivated adenylyl cyclase as an optogenetic tool**

**Authors:** Meenakshi Tanwar^1^, Lohit Khera^2^, Nemneineng Haokip^3^, Rajeev Kaul^2^, Aruna Naorem^3^, and Suneel Kateriya*^1&4^

**Affiliations:**

^1^Department of Biochemistry, University of Delhi South Campus, Benito Juarez Road, New Delhi 110021

^2^Department of Microbiology, University of Delhi South Campus, Benito Juarez Road, New Delhi 110021

^3^Department of Genetics, University of Delhi South Campus, Benito Juarez Road, New Delhi 110021

^4^School of Biotechnology, Jawaharlal Nehru University, New Delhi, India-110067, Telephone: 0091-9312315704

[*skateriya@jnu.ac.in](mailto:*skateriya@jnu.ac.in)

**Figure S1:** Expression of the PACs in HEK-293T cells.

**Figure S2:** Immunoblot analysis of the total CREB in PACs expressing mammalian (HEK-293T) cells.

**Figure S3:** Relative expression of the Cox-2 gene in HEK-293T cells in dark and upon illumination with blue light.

**Figure S4:** Expression of the PACs in *D. discoideum*.

**Figure S5:** Cellular distribution of the GFP-PAC fusion protein in *D. discoideum* transformants.

**Figure S6:** Phenotype of *D. discoideum* Ax2 expressing vector or PACs at 10 h of developmental stage.

**Figure S7:** Developmental phenotype of *D. discoideum* Ax2 (parent strain) in the dark and after illumination with blue light.

**Figure S8:** Immunoblot of the p-CREB in PACs expressing mammalian (HEK-293T) cells.

**Table S1:** Primers used for the cloning of PACs in to pA3M vector for expression in mammalian cells (HEK-293T).

**Table S2:** Primers used for the real time PCR analysis.

**Table S3:** Developmental phenotype of *D. discoideum* Ax2 expressing empty vector or PACs in dark and upon photoactivation with blue light

**Figure S1:**


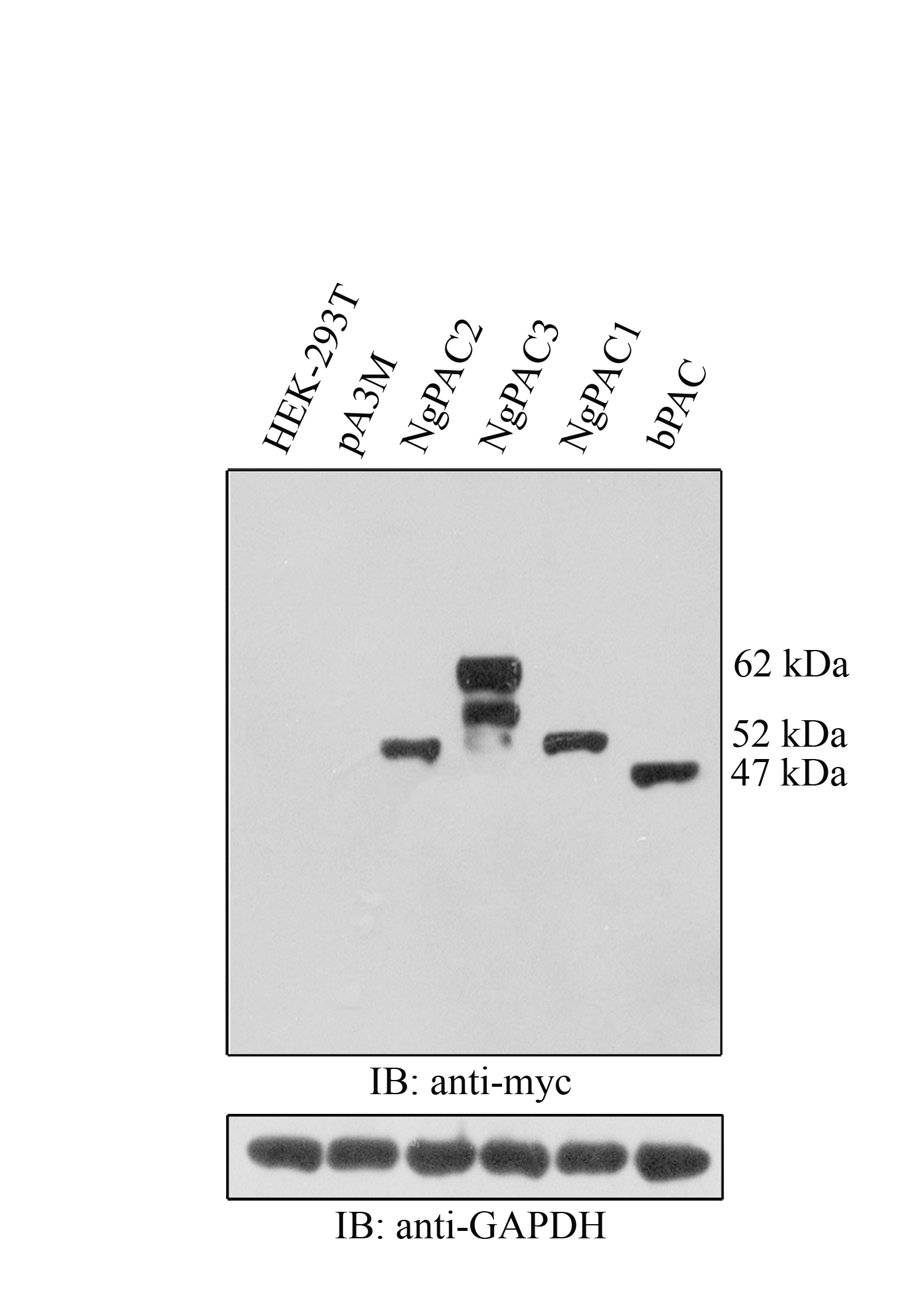


**Figure S1: Expression of the PACs in HEK-293T cells.** Immunoblots confirming the expression of PACs in HEK-293T cells using ployconal anti-myc antibody. Immunoblot using GAPDH antibody (lower panel) serves as a loading control. The sizes of different PACs (NgPAC1- 52 kDa, NgPAC2- 52 kDa, NgPAC3- 62 kDa, and bPAC- 47 kDa) are indicated on right in kDa. Antibody Dilution: anti-myc (1:1000), anti-GAPDH (1:5000).

**Figure S2:**


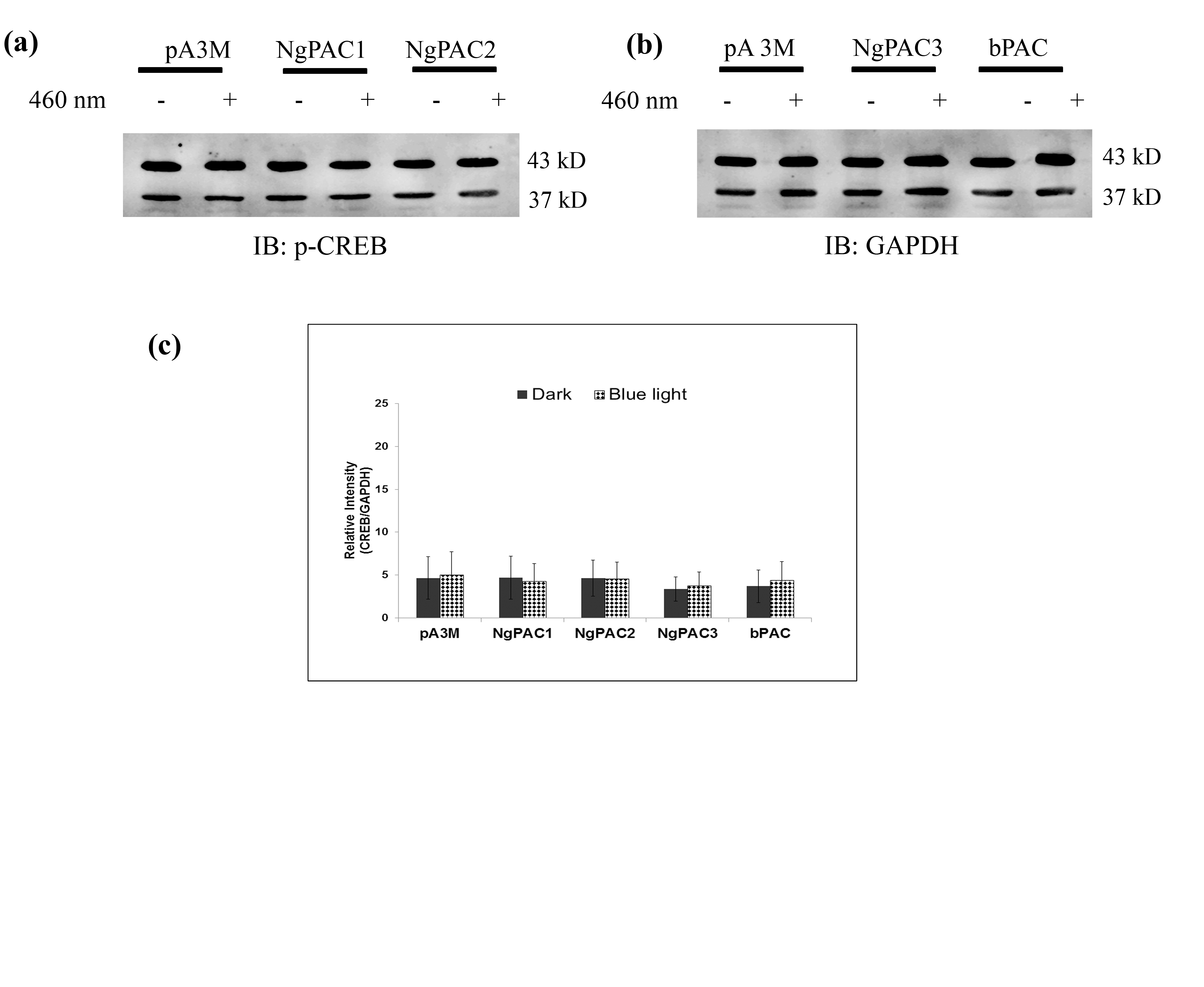


**Figure S2: Immunoblot analysis of total CREB in PACs expressing mammalian (HEK-293T) cells.** Western blot showing total CREB level (upper panel) and GAPDH (lower panel) in the total cell lysate of HEK‐293T cells expressing (a) empty vector pA3M, NgPAC1, NgPAC2, and (b) pA3M, NgPAC3, bPAC, in the dark (‐) and after blue light illumination (+). (c) The relative intensity of CREB:GAPDH in each sample was determined by densitometry using Image J program. The results are the average of the three independent experiments.

**Figure S3:**


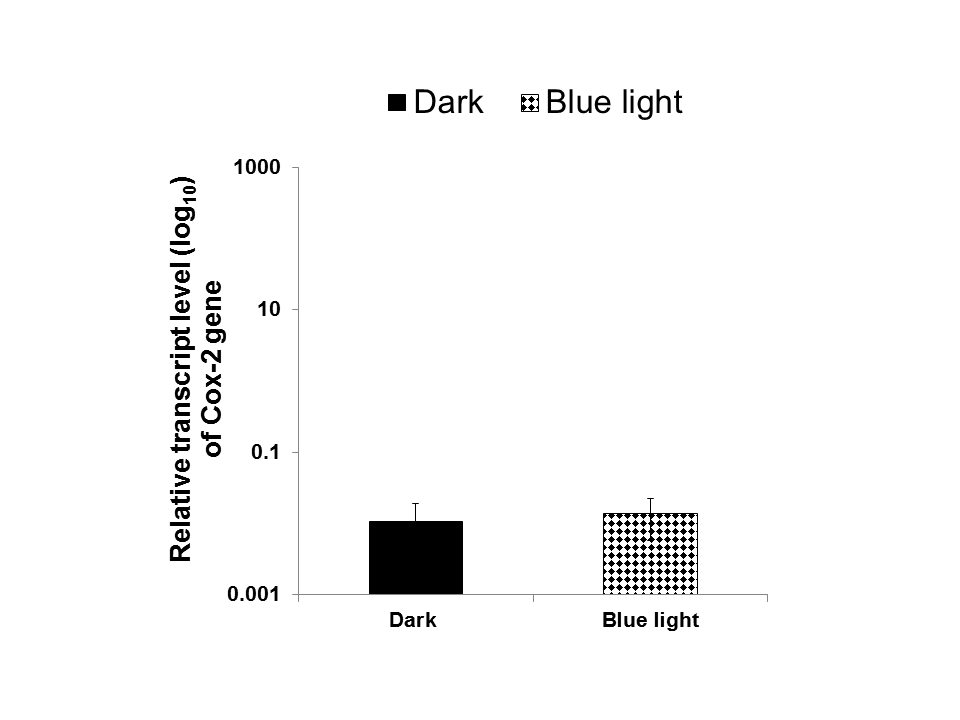


**Figure S3: Relative expression of the Cox-2 gene in HEK-293T cells in dark and upon illumination with blue light.** Cox‐2 mRNA levels (ΔCt values) quantified by qPCR from HEK-293T cells kept in the dark (solid black bar) and after blue light illumination (filled bar). GAPDH mRNA level was used as an internal control. Error bar represents the S.E.M.

**Figure S4:**


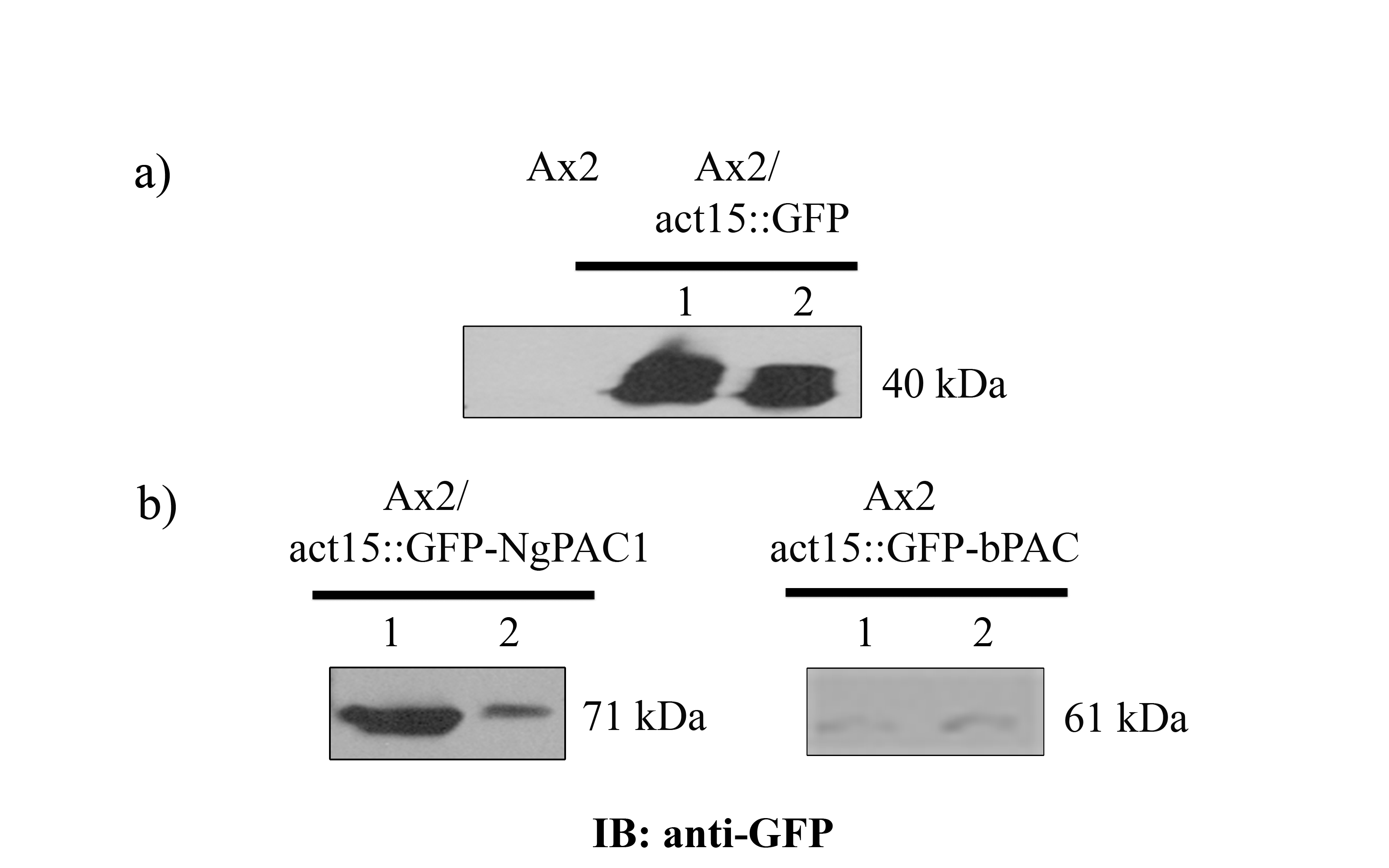


**Figure S4: Expression of the PACs in *D. discoideum*.** Western blotting confirm the expression of GFP, GFP-NgPAC1 and GFP-bPAC fusion in *D. discoideum* Ax2 transformed cells using monoclonal anti-GFP antibody (1:5000 dilution). a) GFP protein in Ax2/act15:: GFP cells lysate and no protein in untransformed Ax2 (wild type) cells lysate. b) GFP-NgPAC1 (71 kDa) and GFP-bPAC (61 kDa) fusion protein in Ax2/act15:: GFP-NgPAC1 and Ax2/act15:: GFP-bPAC cells lysate. Lane 1 and 2 represents the two different clone of respective Ax2 transformed cell.

**Figure S5:**


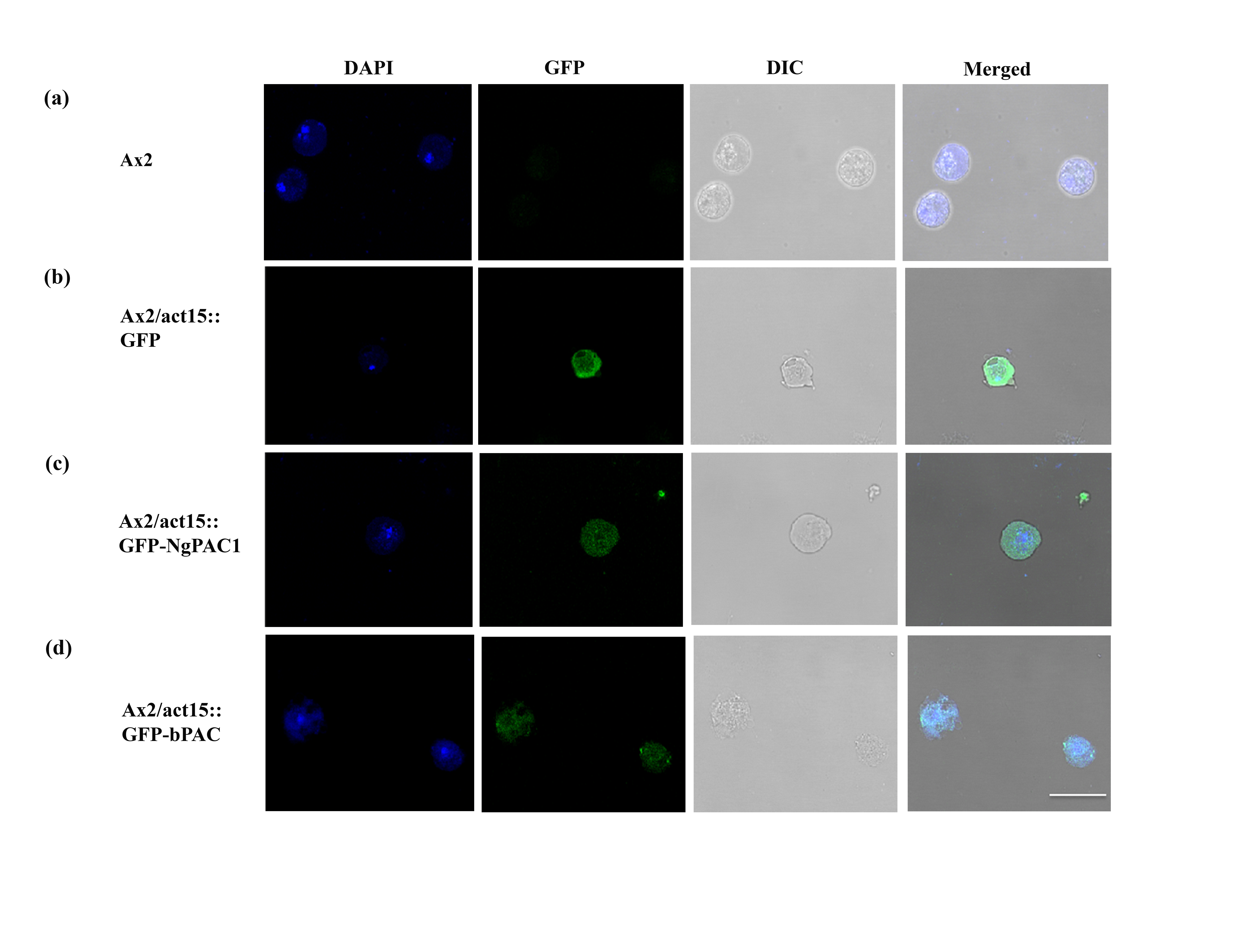


**Figure S5: Cellular distribution of the GFP-PAC fusion protein in *D. discoideum* transformants.** Fluorescence microscopic images of (a) Ax2 untransformed (wild type) cells (control) (b) Ax2/act15:: GFP cells (c) Ax2/act15:: GFP-NgPAC1 cells (d) Ax2/act15:: GFP-bPAC cells, displaying nucleus with blue fluorescence and GFP/GFP-PAC fusion protein with green fluorescence. Left panel: nucleus staining using DAPI, Middle panel: GFP fluorescence and Right panel: DIC image. Extreme right panel: merged image. Scale bar, 20 μm.

**Figure S6:**


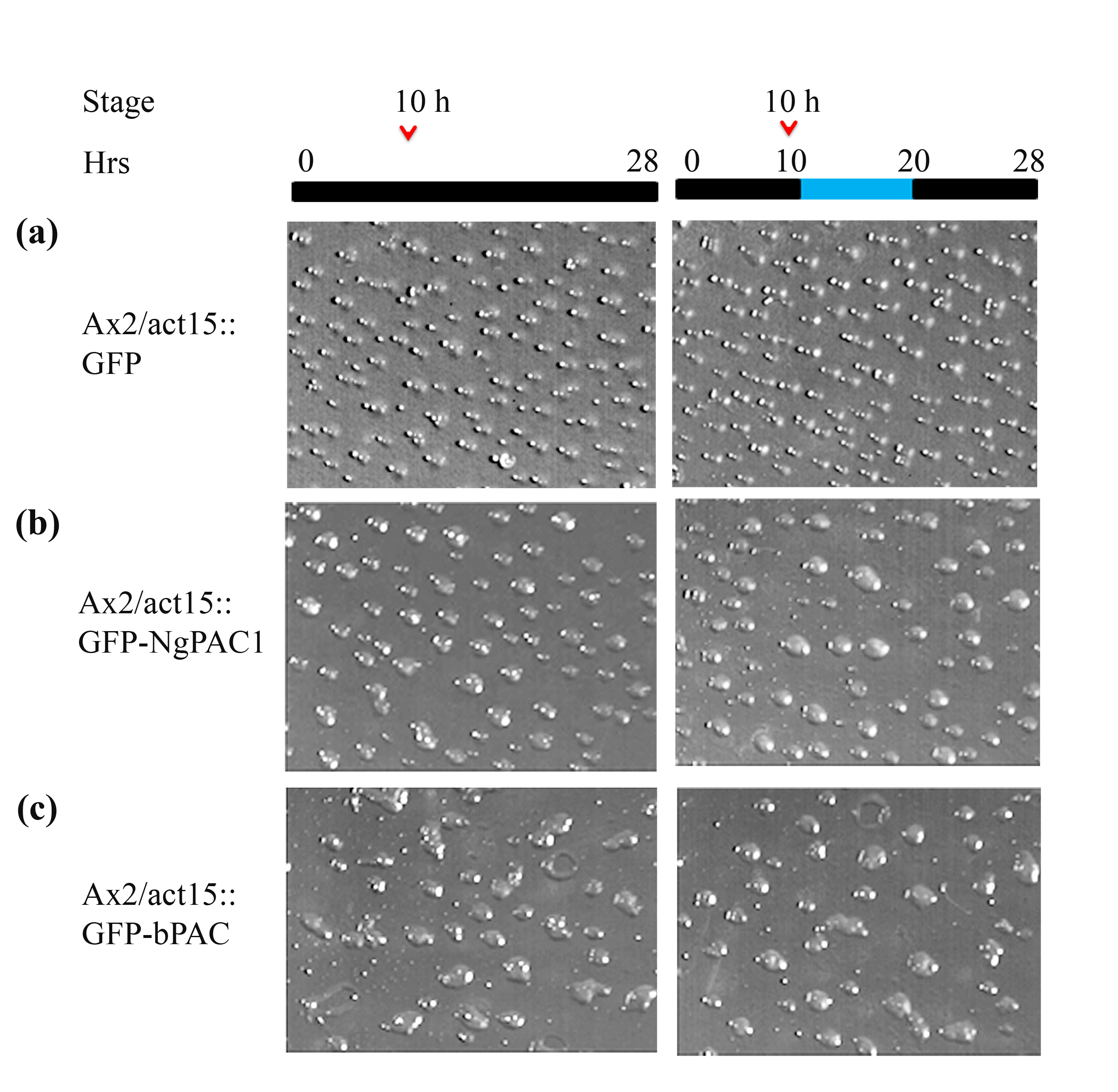


**Figure S6: Phenotype of *D. discoideum* Ax2 expressing vector or PACs at 10 h of development stage.** Development stages of (a) Ax2/act15:: GFP (b) Ax2/act15:: GFP-NgPAC1 (c) Ax2/act15:: GFP-bPAC cells on KK_2_ non-nutrient agar plate. All transformants cells formed mound at 10 h. The cells were plated at a density of 1 x 10^6^ cells/cm^2^. The development stage was chronicled after first 10 h of development (display with red arrow), before irradiation with blue light.

**Figure S7:**


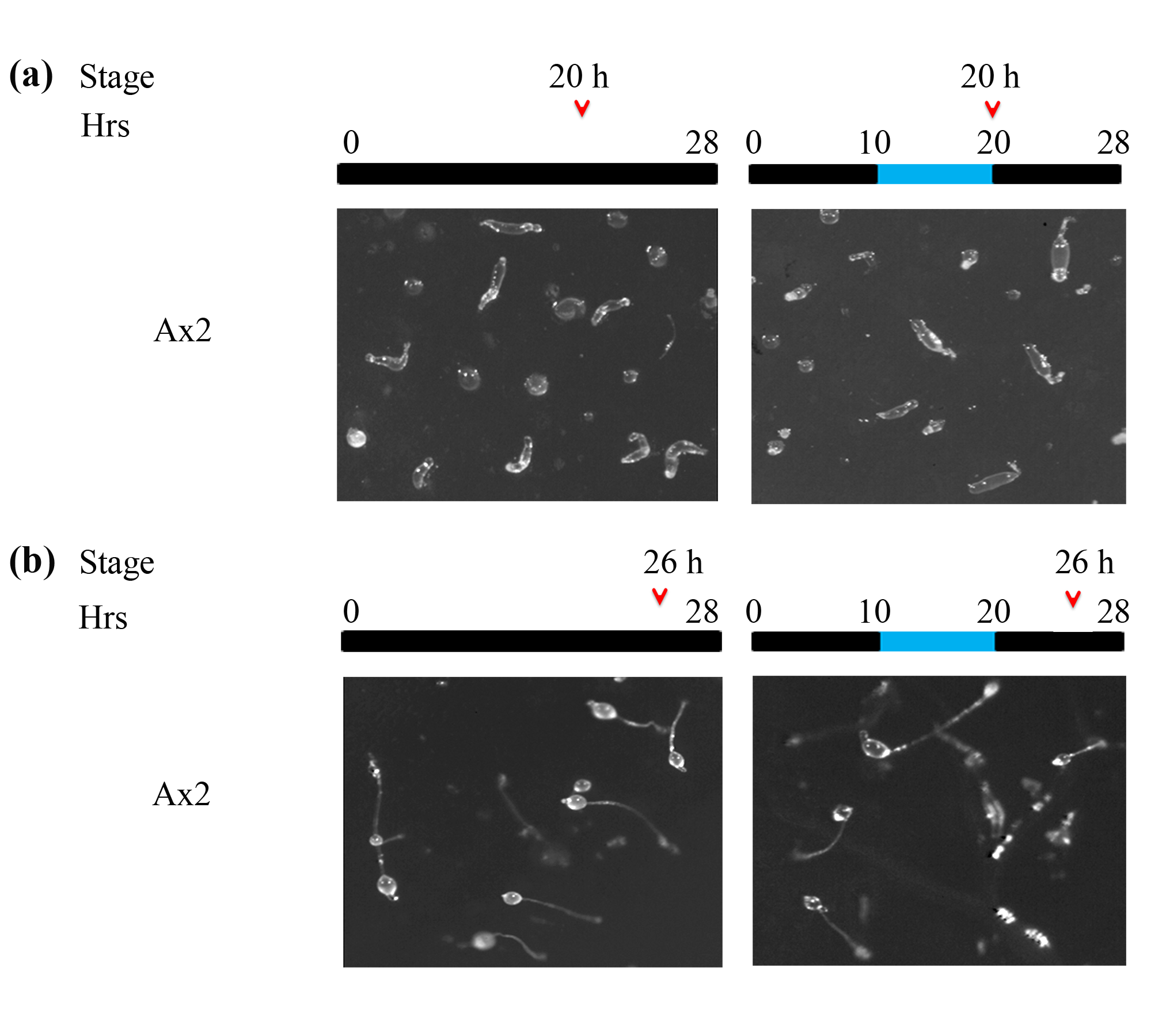


**Figure S7:** **Development phenotype of *D. discoideum* Ax2 (parent strain) in the dark and after illumination with blue light.** Development stages of Ax2 at (a) 20 h of development and (b) 26 h of development on KK_2_ non-nutrient agar plate. The development was carried out in dark and in the presence of blue light after 10 h of development.

**Figure S8:**


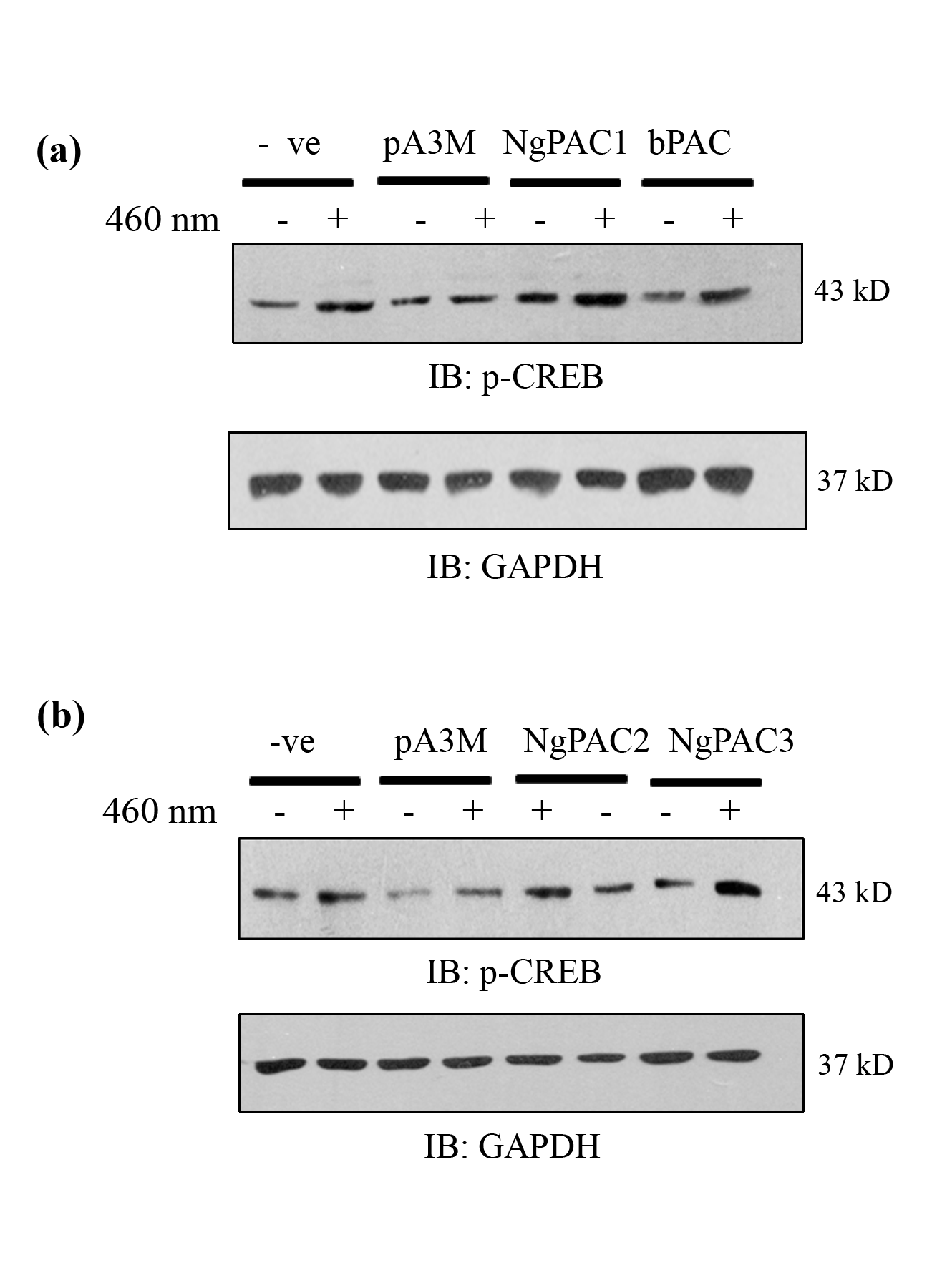


**Figure S8: Immunoblot of p-CREB in PACs expressing mammalian (HEK-293T) cells.** Western blot showing phosphorylated CREB level (upper panel) and GAPDH (lower panel) in the total cell lysate of (a) HEK‐293T cells (-ve), HEK-293T cells expressing empty pA3M vector, NgPAC1, bPAC (b) HEK‐293T cells (-ve), HEK-293T cells expressing empty vector pA3M, NgPAC2 and NgPAC3, in the dark (‐) and after blue light illumination (+).

**Table S1: Primers use for cloning of PACs in to pA3M vector for expression in mammalian cells (HEK-293T).**

| **Oligonucleotide** | **Sequence (5’-3’)** |
| --- | --- |
| NgPAC1 Fwd | ATCGGATCCACCATGAGTAGCATCCAGGAG |
| NgPAC1 Rev | ATTGAATTCGTTCGTACGTCATCTCCAGAC |
| NgPAC2 Fwd | ATCGGATCCACCATGAGTAGCATCACCCA |
| NgPAC2 Rev | GCTGAATTCGTTCGTACGTGAGAATGCTC |
| NgPAC3 Fwd | ATCGGATCCACCATGCCCACAATGAACAAT |
| NgPAC3 Rev | CAGGAATTCGACAGTATTCGGTGAGAGC |
| bPAC Fwd | ATCGGATCCACCATGATGAAACGCCTGGTG |
| bPAC Rev | ACGGAATTCGTTTATCGTTTTCCAGGGTC |

**Table S2: Primers use for the real time PCR analysis**.

| **Oligonucleotide** | **Sequence (5’-3’)** |
| --- | --- |
| GAPDH Fwd | AAGGTGAAGGTCGGAGTCAACG |
| GAPDH Rev | CCTTCTCCATGGTGGTGAAGAC |
| Cox-2 Fwd | GATACT CAGGCAGAGATGATCTACCC |
| Cox-2 Rev | AGACCAGGCACCAGACCAAAGA |

**Table S3: Developmental phenotype of *D. discoideum* Ax2 expressing empty vector or PACs in dark and upon photoactivation with blue light.**

| **Developmental phenotype of *D. discoideum* transformants at 20 hrs development stage** | | | | |
| --- | --- | --- | --- | --- |
| **Transformants** | **Dark** | | **After illumination with blue light** | |
|  | No. of finger or slug (%) | No. of fruiting bodies (%) | No. of finger or slug (%) | No. of fruiting bodies (%) |
| Ax2/act15:: GFP | 73.7±8.75 | - | 68.5±5.75 | -^#^ |
| **Transformants** | **Dark** | | **After illumination with blue light** | |
|  | No. of finger or slug (%) | No. of fruiting bodies (%) | No. of finger or slug (%) | No of fruiting bodies (%) |
| Ax2/act15:: GFP-NgPAC1 | 82.5±6 | -* | 23.75±11.25 | 67.5±19.3*^#^ |
| **Transformants** | **Dark** | | **After illumination with blue light** | |
|  | No. of tipped mound (%) | No. of culminant (%) | No. of tipped mound (%) | No. of culminant (%) |
| Ax2/act15:: GFP-bPAC | 60±17.25 | - | 27.5±12.5 | 61.75±12.25 |
|  | | | | |
| **Developmental phenotype of *D. discoideum* transformants at 26 hrs development stage** | | | | |
| **Transformants** | **Dark** | | **After illumination with blue light** | |
|  | No. of culminant (%) | No. of fruiting bodies (%) | No. of culminant (%) | No. of fruiting bodies (%) |
| Ax2/act15:: GFP | 25.8±4.6 | 71±5.4^#^ | 20±5.9 | 83±7.5 |
| **Transformants** | **Dark** | | **After illumination with blue light** | |
|  | No. of culminant (%) | No. of fruiting bodies (%) | No. of culminant (%) | No. of fruiting bodies (%) |
| Ax2/act15:: GFP-NgPAC1 | 15±17.3 | 72.5±10.5 | 20±9.2 | 82.5±13.8 |
| **Transformants** | **Dark** | | **After illumination with blue light** | |
|  | No. of culminant (%) | No. of fruiting bodies(%) | No. of culminant (%) | No. of fruiting bodies (%) |
| Ax2/act15:: GFP-bPAC | 59.75±7.2 | 23.75± 8.4*^#^ | 18±13.6 | 69.5±12.2* |

The value represents the number of different developmental structures (tipped mound, finger or slug, culminant and fruiting bodies) developed on non-nutrient agar in dark and after illumination with blue light. The percentages are mean ± S.D for two experiments. To determine the effect of photoactivation of PAC on development, number of fruiting bodies formed by Ax2/act15:: GFP-NgPAC1 and Ax2/act15:: GFP-bPAC at 20 h and 26 h respectively were calculated, in dark and after illumination with blue light (Number of multicellular structure = 60-140).

* displayed at the formation of fruiting bodies by Ax2/act15:: GFP-NgPAC1 and Ax2/act15:: GFP-bPAC at 20 h and 26 h indicate a significant difference in dark and after blue light illumination ( *p < 0.05). ^#^ represented at the formation of fruiting bodies by Ax2/act15:: GFP-NgPAC1 and Ax2/act15:: GFP-bPAC at 20 h and 26 h indicate a significant difference with formation of fruiting bodies by Ax2/act15 in light and dark respectively ( *p < 0.05). - indicate no respective structure formed at time point.
